# Nitric Oxide, Reactive Oxygen Species, and Focal Adhesion Kinase Mediate Anoikis Resistance in A375 and SK-MEL-28 Human Melanoma Cells

**DOI:** 10.3390/antiox15060740

**Published:** 2026-06-10

**Authors:** Igor R. do Nascimento, Ana Caroline S. Teodoro, Paulo V. de Sousa, Leticia T. Barboza, Joanderson P. Cândido da Silva, Ricardo C. Cintra, Caroline Alves, Lidia R. De Toledo, Ronaldo J. Carneiro, Luiz S. Longo, Arnold Stern, Hugo P. Monteiro

**Affiliations:** 1 Department of Biochemistry, Center for Cellular and Molecular Therapy—CTCMol, Escola Paulista de Medicina, Universidade Federal de São Paulo, São Paulo 04039-032, Brazil; igor.nascimento@unifesp.br (I.R.d.N.); ana.caroline10@unifesp.br (A.C.S.T.); sousa.vinicius@unifesp.br (P.V.d.S.); letorres.barboza@gmail.com (L.T.B.); joanderson.candido@unifesp.br (J.P.C.d.S.); ricardo.cintra@unifesp.br (R.C.C.); calves@unifesp.br (C.A.); lidia.toledo@unifesp.br (L.R.D.T.); rcarneiro@unifesp.br (R.J.C.); 2 Department of Pharmaceutical Sciences, Campus Diadema, Universidade Federal de São Paulo, São Paulo 09913-030, Brazil; luiz.longo@unifesp.br; 3 Grossman School of Medicine, New York University, New York, NY 10016, USA; sterna01@gmail.com

**Keywords:** melanoma, nitric oxide, NO synthases, anoikis resistance, NADPH oxidase 4, hydrogen peroxide

## Abstract

Melanoma is a highly aggressive and invasive form of skin cancer that arises from the uncontrolled growth of melanocytes. It is characterized by early spread through the lymphatic system and metastasis. The success of metastasis is linked to the ability of melanoma and other cancer cells to resist anoikis, a type of cell death that occurs when cells lose their adhesion to the extracellular matrix. Redox signaling plays an essential role in anoikis resistance. The balance between intracellular levels of nitric oxide (NO) and the reactive oxygen species (ROS) O_2_^−^ and H_2_O_2_ stimulate signaling pathways related to proliferation and survival or cell death. A375 and SK-MEL-28 human melanomas cell lines, representing primary melanoma and lymph node metastatic melanoma cells, respectively, under suspension and adherent culture conditions were used to investigate the redox regulation of anoikis resistance. Both cell lines express the three isoforms of nitric oxide synthases (NOS) and NADPH oxidase 4 (NOX4) as endogenous sources of NO and ROS, respectively. When A375 cells in suspension were treated with the pan-NOS inhibitor L-NAME, their viability decreased. The treatment resulted in a decrease in FAK phosphorylation at Tyr397 and in ERK 1/2 phosphorylation. The expression of FAK, ERK 1/2, β-actin, and α-tubulin were significantly reduced. Treatment with L-NAME led to an increase in the expression of the metalloprotease MMP-9. SK-MEL-28 cells in suspension and treated with the NOX4 inhibitor, GKT36901, exhibited reduced viability. This was accompanied by the inhibition of FAK phosphorylation at Tyr397, ERK 1/2 phosphorylation, and a reduction in the expression of FAK, ERK 1/2, β-actin, and α-tubulin, with a slight elevation in the expression of MMP-9. Migration and invasion were strongly inhibited in A375 cells upon treatment with L-NAME, while treatment with GKT36901 had a marginal effect on the migration and invasion capacities of SK-MEL-28 cells. In summary, melanoma cells employ nitrosative and oxidative stress to shield themselves from anoikis. Nitric oxide was essential for melanoma cells at the primary site for resisting anoikis, while H_2_O_2_ contributed to anoikis resistance in metastatic melanoma cells.

## 1. Introduction

Cutaneous melanoma is particularly aggressive and characterized by the uncontrolled growth and proliferation of melanocytes [1]. Its prevalence has risen significantly over the past three decades, and a total of 354,000 cases were estimated for the period 2022–2025, with disease progression leading to the deaths of 62,600 individuals [2].

The aggressiveness of the disease is related to its rapid development and high metastatic capacity. Malignant melanoma cells migrate through the lymphatic circulation, and in the advanced stages of the disease, metastatic colonization occurs especially in the lung, liver, and brain. The 5-year survival rate for patients with lymph node metastasis is less than 5% [3].

The process of metastasis involves several stages that rely on the ability of tumor cells to break away from the primary tumor, survive in the vascular and lymphatic circulation, and establish and grow in distant organs [4]. A successful metastasis requires cells to resist anoikis, a form of programmed cell death induced after cell detachment from the extracellular matrix (ECM) [5]. Epithelial cells depend on cell–cell and cell–ECM interactions for anchorage and survival [6]. Thus, anoikis is a crucial mechanism for preventing adhesion-independent cellular growth by avoiding the dissemination of these cells in circulation. However, tumor cells can resist anoikis, growing and surviving independently of anchorage [7].

Anoikis resistance has been associated with high expression levels of integrin receptors in cancer cells [8,9,10]. Integrin’s extracellular domain interacts with the ECM, initiating signaling cascades to suppress anoikis [5,11]. Downstream to the integrins are focal adhesion kinase (FAK) and the other components of the focal adhesion complex, Src kinase, the SH2/SH3 adapter protein p130Cas, and the structural protein paxillin, which are involved in signaling pathways responsible for *anoikis* resistance [9]. FAK phosphorylation at Tyr397 and the activation of the focal adhesion complex controls essential cellular processes such as cell adhesion, migration, proliferation, and survival [12,13,14]. FAK phosphorylation triggers the activation of Src kinase and initiates the PI3K-Akt and Raf-MEK-ERK1/2 signaling pathways [15]. Upregulation of these signaling pathways is associated with the epithelial–mesenchymal transition, tumor angiogenesis, and drug resistance [16].

Cancer cells live under chronic oxidative and nitrosative stress conditions, expressing different isoforms of NADPH oxidases (NOX) and nitric oxide synthases (NOS) [17]. Either reactive oxygen species (ROS) or nitric oxide (NO) generated by these particular enzymes stimulate various signaling pathways related to proliferation and survival [18].

Nitric oxide stimulates FAK phosphorylation and its interactions with Src kinase, promoting cell proliferation [19,20]. Exposure of HeLa human cervical cancer cells and murine melanoma cells maintained in suspension for a prolonged period to NO donors at high concentrations results in the activation of Src kinase and anoikis resistance [21]. FAK activation is needed for observing anoikis resistance in HeLa cells, HepG2 human hepatic cancer cells, and MCF-7 human breast cancer cells under nitrosative stress conditions [22]. In melanoma cells, NOS isoform expression is elevated compared with normal melanocytes, and this elevation is directly related to increased metastasis risk [23,24,25].

The expression of NOX4 was related to melanoma progression [26]. Several melanoma cell lines show an increase in NOX4 expression, which is associated with metastasis, while the knockdown of NOX4 results in G2-M cell cycle arrest [26,27,28]. ROS and NO play specific roles during the different stages of melanoma development [29]. However, it is poorly understood how these oxidants are involved in anoikis resistance in melanoma and other types of cancer, and how this process is affected by the stage of cancer development.

This study has investigated the role of NO and ROS in promoting anoikis resistance in human melanoma cells from a primary site and lymph node metastasis. Anoikis resistance is higher in SK-MEL-28 human melanoma cells from lymph node metastasis than in A375 human melanoma cells from the primary site. Nitric oxide is required for protection against anoikis in A375 cells, while H_2_O_2_ is required for protection against anoikis in SK-MEL-28.

Inhibition of NOS sensitizes A375 cells to anoikis, while NOX4 inhibition sensitizes SK-MEL-28 cells to anoikis. Melanoma cells at different stages of development, represented by A375 and SK-MEL-28 cells, use NO and H_2_O_2_, respectively, to overcome *anoikis* and thrive.

## 2. Materials and Methods

### 2.1. Cell Culture Conditions

Tumor cell lines were obtained from the American Type Culture Collection. The human primary tumor melanoma cell line A-375 (ATCC CRL-1619) and human lymph node metastatic melanoma cell line SK-MEL-28 (ATCC HTB-72) were cultured in Dulbecco’s Modified Eagle Medium (DMEM) supplemented with 10% fetal bovine serum (FBS), penicillin (100 mg/mL), and streptomycin (100 mg/mL), and maintained at 37 °C and 5% CO_2_.

### 2.2. Western Blotting

Cell lysates were obtained by incubation with lysis buffer (20 mM HEPES, 150 mM NaCl, 10% glycerol, 1%Triton, 1 mM EGTA, 1.5 mM MgCl_2_ · 6H_2_O, PMSF 1 mM, aprotinin 1 μg/mL and leupeptin 1 μg/mL, NaF 50 mM, NaPyr 10 mM, 2 mM Na_3_VO_4_) for 30 min on ice. Protein concentrations from cell lysates were determined by using the BCA Protein Assay Kit (Thermo Fisher, Waltham, MA, USA). Cell lysates (100 μg/mL) were resolved on 10% SDS-PAGE gels and transferred to PVDF membranes (Amersham™ Hybond™, Little Chalfont, UK). Membranes were incubated in 5% non-fat dry milk for 2 h or 5% BSA for 1 h. After blocking, membranes were incubated with primary antibodies overnight at 4 °C followed by washing and incubation with the appropriate secondary antibody conjugated with horseradish peroxidase. Bands were detected using the Photo-Video Documentation System (Fuji LAS-4000, Tokyo, Japan) and quantified by densitometry using the ImageJ software version 1.54X. Primary antibodies used for Western blot analysis were as follows: rabbit anti-Caspase 3 (1:1000—Cell Signaling, 9662S); rabbit anti-Cleaved Caspase 3 (1:1000—Cell Signaling, 9664S); rabbit anti-FAK-1 (1:1000—Cell Signaling, 3285S); rabbit anti-Alpha-tubulin (1:1000—Cell Signaling, 2144S); rabbit anti-pan-NOS (1:1000—Cell Signaling, 2977); rabbit anti-GAPDH (1:1000—Cell Signaling, 4874); rabbit anti-Phospho-FAK (Y397) (1:1000—Millipore, EP2106Y, 04-974); rabbit anti-NOX4 (1:1000—Abcam, ab109225); mouse anti-β-actin (1:5000—Sigma Aldrich, A1978); rabbit anti ERK1/2 (1:1000—Cell Signaling,9102); rabbit anti phospho-ERK1/2 (1:1000—Cell Signaling, 9101); rabbit anti-MMP9 (1:1000—Abcam EP1254). Secondary antibodies were horseradish peroxidase (HRP)-conjugated NA934 (Cytiva, Amersham, Ann Arbor, MI, USA).

### 2.3. Generation of A375 Cells Knockdown for NOS2

To generate stable A375 cells knockdown for NOS2, short hairpin RNA (shRNA) sequences against the *NOS2* gene and the Scramble control (TR30021), were obtained from (Origene™ Technologies, Rockville, MD, USA). The sequence used was: iNOS _ SH construct: GCTATCGAATTTGTCAACC (HC137047). HEK 293T cells plated in 24-well plates at 2  ×  10^4^ cell density were kept in DMEM high glucose medium plus 10% FBS. After plating, culture medium was replaced with fresh DMEM high glucose plus 5% FBS without antibiotics. Viral transfection was performed in HEK 293T cells using the transfection kit TLP5910 (Dharmacon^®^, Lafayette, CO, USA). Viral particles (Origene™ Technologies, Rockville, MD, USA), were kept at −80 °C. A375 cells were infected with 6 µg of viral particles (sh_iNOS or Scramble) and 8 µg/mL polybrene (Origene™ Technologies, Rockville, MD, USA). After 72 h, knockdown was confirmed by fluorescence microscopy followed by treatment with 4 µg/mL puromycin (Sigma-Aldrich, St. Louis, MO, USA) for selection. After 48 h of puromycin selection, A375 NOS2 GFP-positive cells were sorted using a FACS Aria Cell Sorter Flow cytometer (BD).

Knockdown efficiency was evaluated by Western blot analysis of the expression levels of NOS2.

### 2.4. Generation of A375 Cells Knockdown for NOS3

The promoter region and sequences adjacent to the transcription start site (TSS) of the NOS3 gene were analyzed, and two pairs of sgRNAs were designed between −50 and +300 bp relative to the TSS. This positioning enhances CRISPRi efficiency by blocking RNA polymerase II progression, thereby reducing gene transcription [30]. sgRNAs were designed using CRISPOR, prioritizing guides with ≥4 mismatches and at least two mismatches in the seed region to minimize off-target effects [31] (Table 1).

#### 2.4.1. Vectors Construction

Vectors based on dCas9-KRAB, which enable transcriptional repression without introducing DNA breaks, were used. sgRNAs were cloned into the vectors by digestion with BbsI or BsmBI, followed by ligation with T4 DNA ligase. Transformation was performed by electroporation, and colonies grown on LB-agar supplemented with ampicillin were screened by PCR. Positive colonies containing the correct sgRNA insert (103 bp) were expanded and plasmids were purified using a Miniprep protocol.

#### 2.4.2. Cell Transfection and Selection

Cells were seeded in 6-well plates and transfected with the dCas9-KRAB-sgRNA vector using Fugene^®^HD (Promega, Madison, WI, USA) in Opti-MEM^®^ medium (Thermo Fisher, Waltham, MA, USA). After 48 h, puromycin selection was applied to generate stable pools of transfected cells. Knockdown efficiency was evaluated by Western blot analysis of the expression levels of NOS3.

### 2.5. Anoikis Assay

Tissue culture six-well plates were coated with 25 mg/mL poly-HEMA (Sigma-Aldrich, St. Louis, MO, USA) diluted in 99% ethanol (MerckKGaA, Darmstadt, Germany) to prevent cell adhesion to a growth-permissive surface (experimental anoikis). Coated six-well plates were left to evaporate for 48 h in a laminar flow sterile cabin at room temperature. After evaporation, the plates were washed with PBS. Cells in adherent conditions and cultivated in DMEM (Vitrocell Embriolife, Campinas, SP, Brasil) supplemented with 10% fetal bovine serum (FBS) (Thermo-Fisher, Waltham, MA, USA) were trypsinized and a suspension of these cells in serum-starved DMEM was seeded in poly-HEMA-coated plates. Cells were maintained in the absence of anchorage at 37 °C and 5% CO_2_ for a 48 h period, in the presence or absence of increasing concentrations of the NO donor DETANonoate (Detano) (Cayman, Ann Arbor, MI, USA) or the Pan-NOS inhibitor, L-Nitro-Arginine-Methyl Ester (L-NAME) (Sigma Aldrich, St. Louis, MO, USA) or the NOX4 inhibitor GKT136901 (Cayman, Ann Arbor, MI, USA). Cells were collected and used in different assays.

### 2.6. Estimation of Intracellular Production of NO

Cells were grown in a 12-well plate for 24 h in DMEM medium supplemented with 10% FBS (complete medium). After this period, the medium was replaced with a DMEM medium without FBS (serum restriction) and maintained in these conditions for 48 h. Cells were then incubated with 5 μM DAF-2 DA (Sigma Aldrich, St. Louis, MO, USA) for 30 min at 37 °C in the dark. Cells were detached and cell-associated fluorescence was analyzed in a Becton-Dickinson FACS Canto II flow cytometer, with a laser emitting excitation at 495 nm and emission at 515 nm. Results are expressed as DAF-2-derived fluorescence intensity median values obtained for cells under serum restriction.

### 2.7. Estimation of H_2_O_2_ Production

Cellular H_2_O_2_ production was assessed by Amplex UltraRed (Molecular Probes^®^—Invitrogen, Eugene, OR, USA). SK-Mel-28 and A375 cells in non-adherent and adherent conditions were seeded in DMEM 10% FBS on a 100 mm cell culture dish (1 × 10^6^/dish). Cells were washed with HBSS without phenol red, Ca^2+^, and Mg^2+^ (Sigma-Aldrich, St. Louis, MO, USA). Cells were trypsinized and centrifuged at 1,500 rpm for 10 min at 22 °C. After this step, 2.5 × 10^4^ cells were used for the assay. The assay was carried out in the absence of direct light in an opaque 96-well plate. For the reaction, 100 μL of the reagent mixture (0.1 mM Amplex Red and 1 U/mL peroxidase-HRP) and 20 μL of stop reagent Amplex™ Red/UltraRed Stop Reagent, (Molecular Probes^®^—Invitrogen, Eugene, OR, USA) were added to each well. Next, the reaction was incubated at 37 °C for 10 min in a spectrofluorometer Spectra Max Gemini (Molecular Devices, San Jose, CA, USA). After incubation, the cell suspension (in a volume of 100 μL) was added to the wells, and the fluorescence was measured with 560/590 nm excitation/emission for 1 h in 1 min intervals. Readings were taken at 37 °C. The H_2_O_2_ production was calculated by a calibration curve with known concentrations of H_2_O_2_.

### 2.8. Cell Viability Assay

Cells were seeded (1 × 10^5^) in a 24-well plate and maintained in a complete medium for 24 h. After this time, the medium was replaced with DMEM supplemented with 0.25% FBS. After that, cells were mixed with 0.4% Trypan Blue solution (Thermo-Fisher, Waltham, MA, USA). Colored (non-viable) and dye-excluding (viable) cells were counted on an automated cell counter Countess 3 (Thermo-Fisher, Waltham, MA, USA).

### 2.9. Annexin V/FITC Propidium Iodide Assay

Cells in non-adherent and adherent conditions were exposed to L-NAME (25 mM) for 48 h. After this period, cells were washed with PBS and incubated with FITC Annexin V and Propidium Iodide (PI) (Molecular Probes^®^—Invitrogen, Eugene, OR, USA) in binding buffer (10 mM HEPES, 140 mM NaCl, 2.5 mM CaCl_2_, pH 7.4) for 30 min at room temperature. Cells were washed with PBS, and cell-associated fluorescence was analyzed in a Becton-Dickinson FACS Canto II flow cytometer.

### 2.10. Cell Aggregation and Reattachment Assays

Cells at a density of 1  ×  10^6^ cells per well were plated for 48 h at 37 °C, 5% CO_2_ in non-adherent conditions. Cells maintained in non-adherent conditions were exposed to L-NAME (25 mM) or H_2_O_2_ (Merck, KGaA, Darmstadt, Germany) (500 µM, 1 mM, 2 mM and 3 mM) or GKT136901 (320 µM). Cells formed aggregates that were examined under an inverted phase-contrast microscope and photographed.

To analyze cell reattachment to a permissive surface, cells were collected and washed after being in suspension for 48 h in the presence of H_2_O_2_. Cells were suspended in DMEM supplemented with 5% FBS and seeded in six-well plates that have an adherent surface and were maintained in these conditions for an additional 24 h. The number of cells reattached and their morphological changes were examined under an inverted phase contrast microscope. The percentage of cells that reattached was determined by counting viable cells in an automated cell counter Countess 3 (Thermo-Fisher, Waltham, MA, USA).

### 2.11. Migration and Invasion Assays

Cell migration and invasion were evaluated using Transwell^®^ chambers with 8 μm pore-size polycarbonate membranes (Corning, Corning, NY, USA), as previously described [32]. Cells were trypsinized, counted, and resuspended in serum-free medium. A total of 1 × 10^5^ cells were seeded into the upper chamber, while the lower chamber was filled with complete medium containing 10% fetal bovine serum (FBS) as a chemoattractant. For invasion assays, the inserts were pre-coated with Matrigel^®^ (Corning, Corning, NY, USA), diluted in serum-free medium, and allowed to polymerize at 37 °C. Migration assays were performed without Matrigel^®^ coating. Cells were incubated for 96 h at 37 °C in a humidified atmosphere with 5% CO_2_. After incubation, non-migrated/non-invaded cells were removed from the upper surface using a cotton swab. Cells that had migrated or invaded the lower surface of the membrane were fixed with 4% paraformaldehyde, stained with 0.1% crystal violet, and quantified by manual counting under a light microscope in randomly selected fields. Experiments were performed in triplicate, and results were expressed as mean ± standard deviation (SD).

### 2.12. Computational Tools for Analysis of Protein–Protein Interactions

Using the Gene Ontology (GO) and Kyoto Encyclopedia of Genes and Genomes (KEGG) pathway enriched analysis, we constructed a protein–protein interaction (PPI) network involving six genes: *NOS3*, *NOX4*, *PTK2 (FAK)*, *TUBA1A (α-tubulin)*, *ACTB (β-actin)*, *SRC*, *MAPK3* (ERK1), *MAPK1* (ERK2), and *MMP9* using the STRING database http://string-db.org/ (accessed on 27 April 2026). In the network, proteins are represented by nodes and their interactions by edges. The PPI relationships were analyzed using the STRING database. False discovery rate (FDR) and Strength values were employed to determine the relevance of the interactions.

### 2.13. Statistical Analysis

Statistical analysis was performed using Prism 8 software (GraphPad Software, San Diego, CA, USA). Comparison among multiple groups was performed by one-way analysis of variance (ANOVA), followed by Bonferroni multiple comparison tests. Comparisons between the two groups were performed by *t*-test. Results are described as the mean ± SD. Results are the mean of at least three separate experiments in each group.

## 3. Results

### 3.1. Anoikis in A375 and SK-MEL-28 Human Melanoma Cell Lines

**Figure 1:**
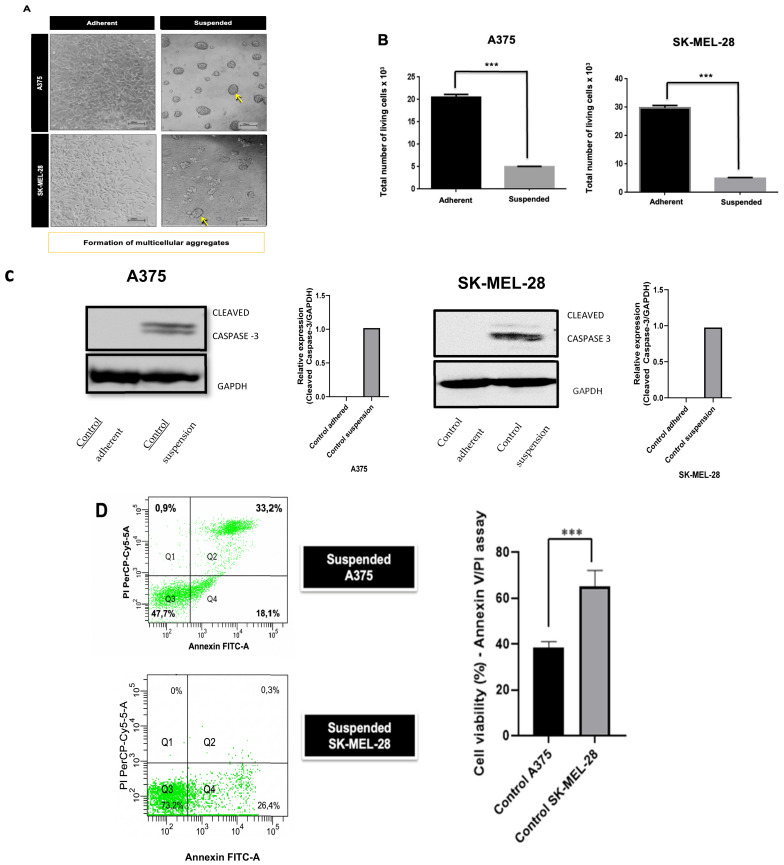
Standardization of the *anoikis*-induced cell death model. (**A**) The image shows A375 and SK-MEL-28 cells cultured in both adherent and suspended conditions for 48 h. The suspended condition was created using poly-HEMA treatment, as described in the Section 2. Yellow arrows show the multicellular aggregates formed by suspended cells. The image was obtained using an inverted microscope with a 100× objective lens. (**B**) Cell viability assay for both cell lines maintained in adherent and suspended conditions for 48 h. The number of dead cells was determined by the Trypan Blue exclusion assay. Values are representative of three independent experiments. (**C**) Western blot analysis was performed to evaluate the expression of cleaved caspase-3 in A375 and SK-MEL-28 cells maintained in suspension for 48 h in serum-free DMEM. The blot images shown are representative of three independent experiments. (**D**) Annexin V/PI labeling was performed on A375 and SK-MEL-28 cells maintained in suspension for 48 h in serum-free DMEM. Cells were labeled with Annexin V (Q4, indicating apoptosis), propidium iodide (PI) (Q1, indicating late apoptosis/necrosis), or both (Q2). Non-labeled cells (Q3) represent viable cells. Values are representative of three independent experiments. Difference between means (B − A) ± SEM 26.53 ± 4262. 95% confidence interval 14.70 to 38.37. R squared (eta squared) 0.9064. (*** *p* < 0.05).

A375 (primary site) and SK-MEL-28 (lymph node metastasis) that were maintained in suspension for 48 h in DMEM serum-free medium partially lost their viability, accompanied by the formation of multicellular aggregates (Figure 1A,B). Adherent cells maintained in the same experimental conditions neither detached from the culture plate nor lost their viability (Figure 1B).

The Annexin V and Propidium Iodide (PI) apoptosis detection assay confirmed the findings, indicating that both cell lines lost their viability to a different degree. The expression of cleaved-caspase-3, which is associated with anoikis occurrence [33], was observed only in cells maintained in suspension (Figure 1C). After being kept in suspension for 48 h, 47.7% of A375 cells and 73.2% of SK-MEL-28 cells remained viable during this period (Figure 1D). Survival rates determined for the two cell lines kept in suspension ranged from 60.6 to 73.2% for SK-MEL-28 cells, and 41.4 to 47.7% for A375 cells.

In adherent A375 cells maintained in DMEM serum-free medium for 48 h, viable cells were 97.4% of the total population. In adherent SK-MEL-28 cells maintained in the same conditions, viable cells were 87.7% of the total population (Figure S1).

### 3.2. Anoikis Resistance in A375 Cells Is Mediated by Nitric Oxide

**Figure 2:**
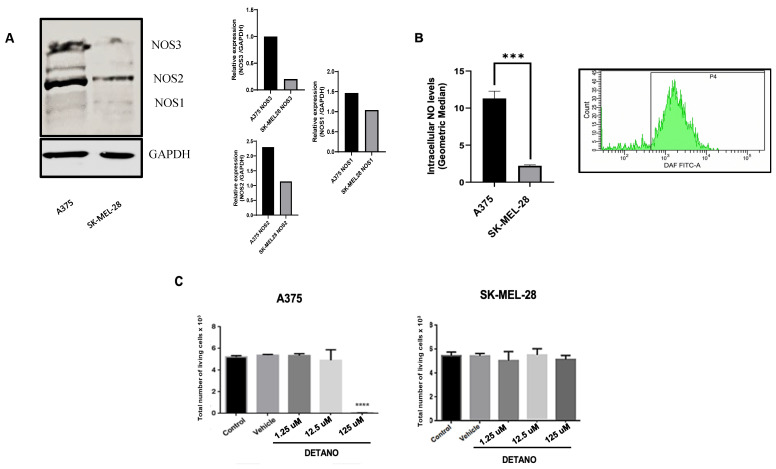
Nitric oxide sources and nitrosative stress in A375 and in SK-MEL-28 cells. (**A**) Western blot analysis was conducted to assess the expression of NOS1, NOS2, and NOS3 in A375 and SK-MEL-28 melanoma cells. The blot images shown are representative of three independent experiments. (**B**) Determination of intracellular levels of NO in suspended A375 and SK-MEL-28 cells using the cell-permeable fluorophore DAF-2 DA. The DAF-2 DA fluorescence intensity is directly related to NO intracellular levels (*** *p* < 0.05). (**C**) Effects of increasing concentrations of Detano on cell viability of A375 and SK-MEL-28 cells kept in suspension for 48 h in serum-free DMEM. Results are expressed as mean ± SD from three independent experiments (*n* = 3) (**** *p* < 0.01).

Expressions of NOS3, NOS2, and NOS1 were detected in A375 and in SK-MEL-28 cells using an anti-pan-NOS antibody. Higher expression levels of NOS2 and NOS3 were found in A375 cells compared to SK-MEL-28 cells. Low expression levels of NOS1 were found in both cell lines (Figure 2A). The intracellular NO concentration measured in both cell lines kept in suspension was higher in A375 cells compared to SK-MEL-28 cells (Figure 2B).

Suspended A375 and SK-MEL-28 cells treated with 1.25 and 12.5 μM of Detano showed no changes in cell viability, while treatment of A375 cells with 125 μM Detano resulted in a complete loss of viability in A375 cells, but not in SK-MEL-28 cells (Figure 2C).

**Figure 3:**
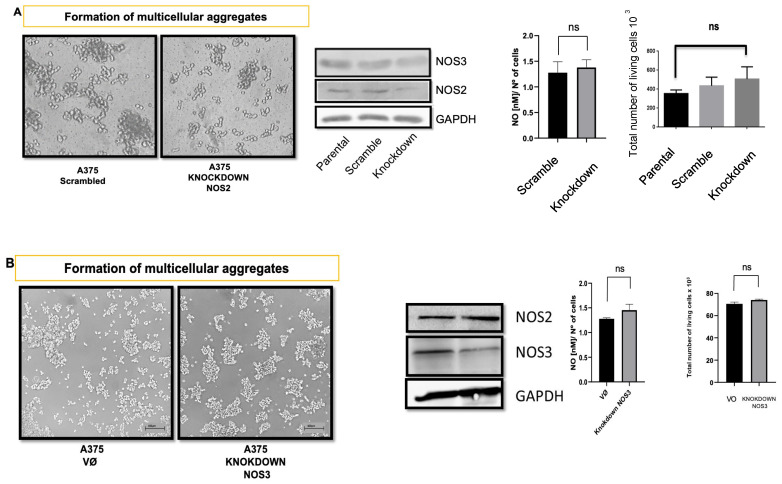
The role of nitric oxide in anoikis resistance of A375 cells. (**A**) Formation of multicellular aggregates, Western blot analysis of NOS3 and NOS2, endogenous production of NO, and the Trypan Blue exclusion assay for A375 cells knockdown for NOS2 kept in suspension in serum-free DMEM for 48 h. Results shown are from three independent experiments (*n* = 3). (**B**) Formation of multicellular aggregates, Western blot analysis of NOS3 and NOS2, endogenous production of NO, and the Trypan Blue exclusion assay for A375 cells knockdown for NOS3 kept in suspension in serum-free DMEM for 48 h. The images shown are representative of three independent experiments. Knockdown cell lines were obtained as described in the Section 2.

A specific shRNA was employed to knock down NOS2 in A375 cells. After maintaining these cells in suspension for 48 h, no significant reduction was observed in cell viability, the formation of multicellular aggregates, or NO production, despite a 60% reduction in NOS2 expression compared to parental cells (Figure 3A).

CRISPR/Cas9 technology was used to obtain a NOS3-knockdown variant of A375 cells. These cells presented an approximate 60% reduction in NOS3 expression. A375 cells in suspension showed no significant reduction in viability, the formation of multicellular aggregates, and NO production as compared to parental cells (Figure 3B). These findings suggest that the NOS isoforms may compensate for each other as intracellular sources of NO in A375 cells in suspension.

**Figure 4:**
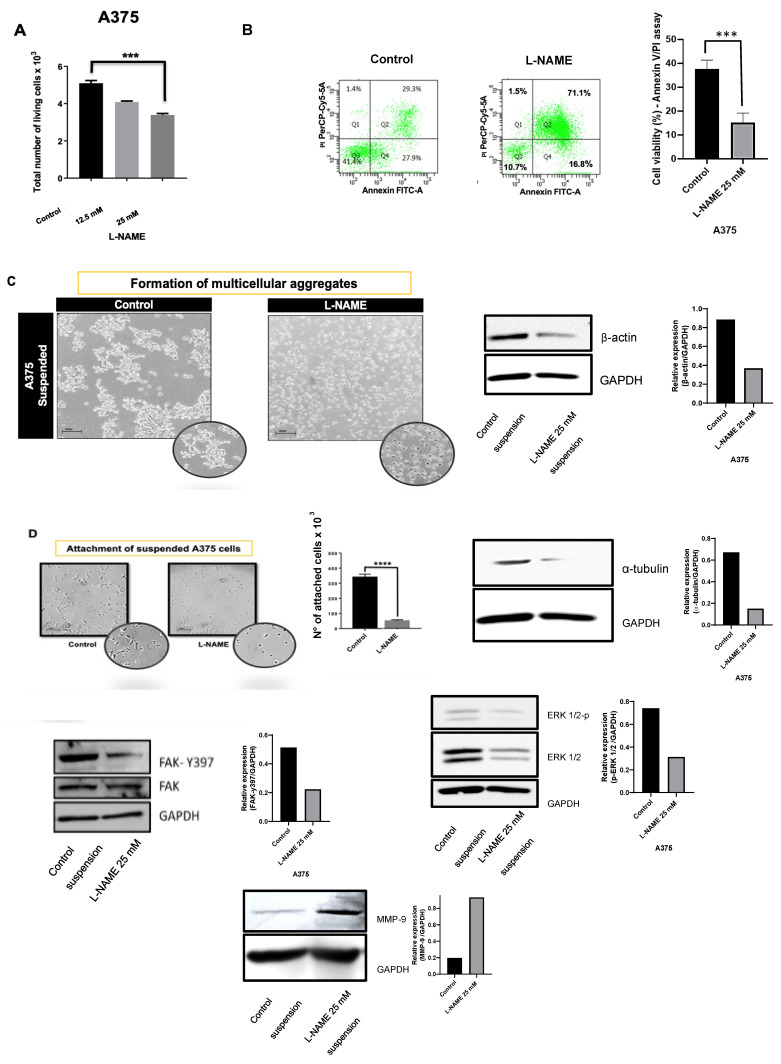

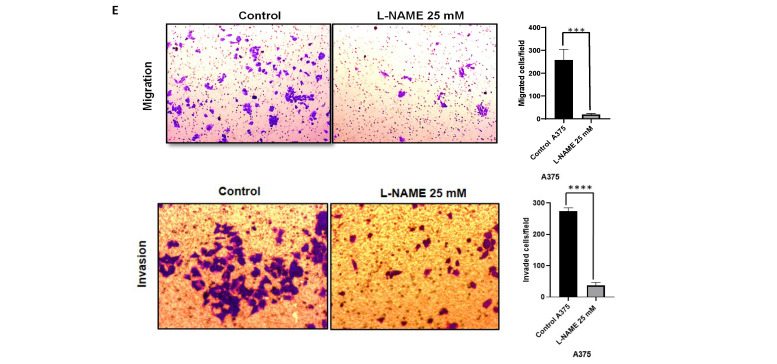
Effects of the treatment of L-NAME on anoikis resistance of A375 cells. (**A**) Cell viability assay for A375 cells maintained in suspended conditions for 48 h and treated with increasing concentrations of L-NAME. The number of dead cells was determined by the Trypan Blue exclusion assay (*n* = 3). (**B**) Annexin V/PI labeling was performed on A375 cells maintained in suspension for 48 h in serum-free DMEM, treated or not with L-NAME 25 mM. Cells were labeled with Annexin V (Q4, indicating apoptosis), propidium iodide (PI) (Q1, indicating late apoptosis/necrosis), or both (Q2). Non-labeled cells (Q3) represent viable cells (*n* = 3), (*** *p* < 0.05). (**C**) Formation of multicellular aggregates and Western blot analysis of β-actin expression levels in suspended A375 cells treated with L-NAME for 48 h (*n* = 3). (**D**) Attachment assay of suspended A375 cells treated with L-NAME (25 mM). Representative images of attached A375 cells after 48 h of treatment were obtained using an inverted microscope (10× objective lens). The number of attached viable A375 cells was determined using the Trypan Blue exclusion assay (*n* = 3). Expression levels of α-tubulin, FAK, FAK activation (p-FAK Tyr397), ERK phosphorylation (p-ERK), and MMP-9 were determined by Western blot analysis. The blot images shown are representative of three independent experiments. (**E**) Cell migration and invasion assays were performed using Transwell chambers with 8 μm pore-size membranes. After the experimental period, cells that migrated or invaded to the lower surface of the membrane were fixed, stained with crystal violet, and quantified. Representative images from three independent experiments are shown. Results are expressed as mean ± SD from three independent experiments. Significance for the migration assay (*** *p* < 0.05). Significance for the invasion assay (**** *p* < 0.01).

Increasing concentrations of L-NAME had an impact on the viability of A375 cells kept in suspension for 48 h (Figure 4A). After analyzing the apoptosis rate in A375 cells in suspension and treated with L-NAME (25 mM), only 10.7% of the cells survived in comparison to 41.4% of the untreated cells (Figure 4B).

A375 cells in suspension formed multicellular aggregates. Treatment with L-NAME inhibited both the formation of these aggregates and the expression of β-actin. Cell attachment was also inhibited after L-NAME treatment (Figure 4C). Western blot analysis indicated that treatment of A375 cells in suspension with 25 mM of L-NAME reduced the expression of α-tubulin, FAK, Tyr397 phosphorylation of FAK, ERK phosphorylation, and increased the expression of the matrix metalloprotease MMP-9 (Figure 4D). L-NAME inhibits the attachment of A375 cells maintained in suspension. These molecular alterations were accompanied by a significant reduction in the migratory capacity of A375 cells, as well as decreased invasive potential, indicating that L-NAME inhibits key processes associated with cell motility and invasion (Figure 4E).

### 3.3. Anoikis Resistance in SK-MEL-28 Cells Is Mediated by Hydrogen Peroxide

**Figure 5:**
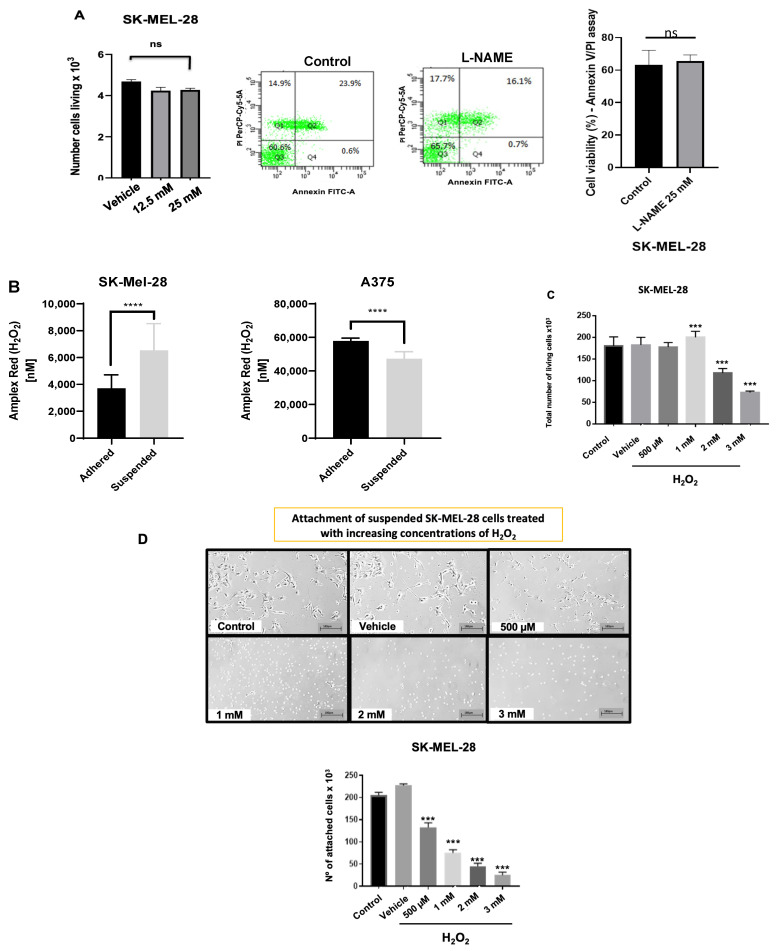
Hydrogen peroxide and anoikis resistance in suspended SK-MEL-28 cells. (**A**) Cell viability assay for SK-MEL-28 cells maintained in suspension for 48 h and treated with increasing concentrations of L-NAME. The number of dead cells was determined using the Trypan Blue exclusion assay (*n* = 3). Annexin V/PI labeling was performed on SK-MEL-28 cells maintained in suspension for 48 h in serum-free DMEM, treated or not with L-NAME (25 mM). Cells were labeled with Annexin V (Q4, indicating apoptosis), propidium iodide (PI) (Q1, indicating late apoptosis/necrosis), or both (Q2). Non-labeled cells (Q3) represent viable cells (*n* = 3). (**B**) Production of H_2_O_2_ in SK-MEL-28 and A375 cells cultured in adherent and suspended conditions for 48 h. The endogenous concentrations of H_2_O_2_ were determined by using the Amplex UltraRed assay. Results shown were obtained in three independent experiments, (**** *p* < 0.01). (**C**) SK-MEL-28 cells kept in suspension were treated with increasing concentrations of H_2_O_2_. The number of dead cells was determined using the Trypan Blue exclusion assay. Results shown were obtained in three independent experiments. (**D**) The attachment assay was performed on suspended SK-MEL-28 cells treated with increasing concentrations of H_2_O_2_. A representative image of the attached SK-MEL-28 cells is shown after 48 h of H_2_O_2_ treatment. The images were captured using an inverted microscope with a 10× objective lens. The counting of attached SK-MEL-28 cells was conducted using the Trypan Blue exclusion method in three independent experiments, and the results showed statistical significance (*** *p* < 0.05).

SK-MEL-28 cells in suspension showed a viability of 60.6%. Unlike A375 cells, SK-MEL-28 cells retained their viability when treated with 25 mM L-NAME, with 65.7% of the cells remaining viable (Figure 5A). This suggests that SK-MEL-28 cells when compared to A375 cells, are more resistant to anoikis-induced cell death, and indicates that this resistance is not dependent on the intracellular NO concentration.

SK-MEL-28 cells in suspension generated higher levels of H_2_O_2_ compared to adherent cells, while A375 cells exhibited a moderate decrease in endogenous H_2_O_2_ levels when they were maintained in suspension as compared to adherent cells (Figure 5B).

The viability of SK-MEL-28 cells in suspension was unaffected by treatment with 500 μM or 1 mM H_2_O_2_. However, exposure to higher concentrations (2 mM and 3 mM) resulted in an extensive loss of cell viability (Figure 5C) and impairment of the ability to reattach to a growth-permissive surface (Figure 5D).

**Figure 6:**
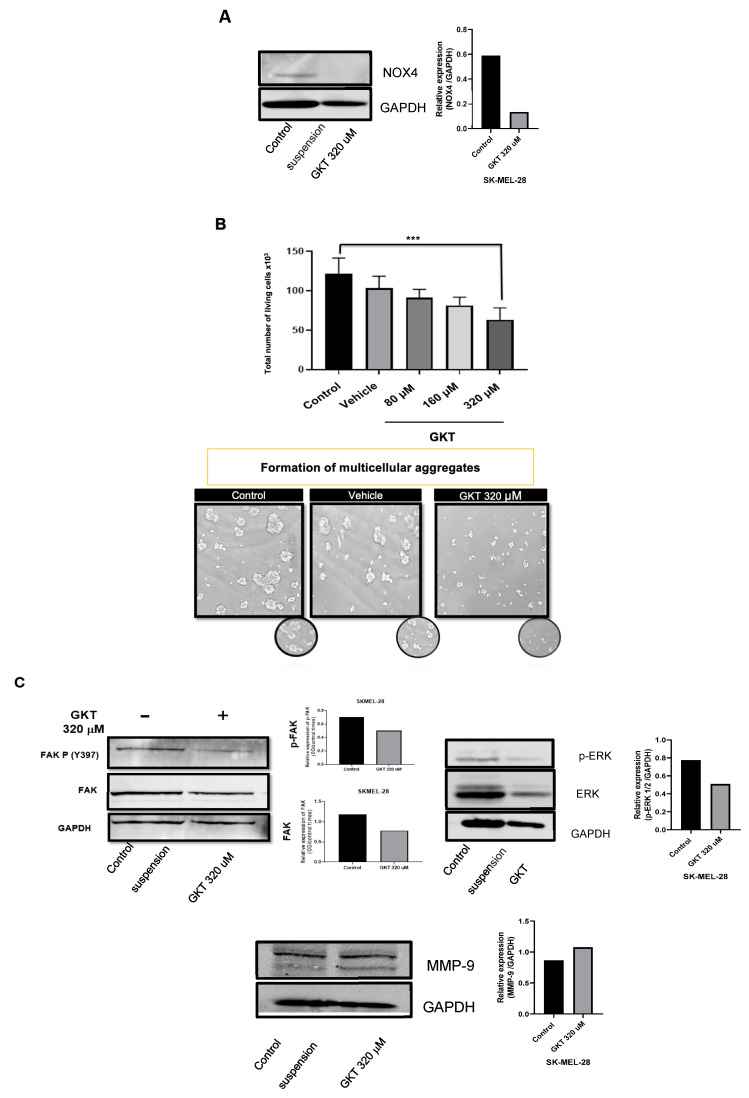

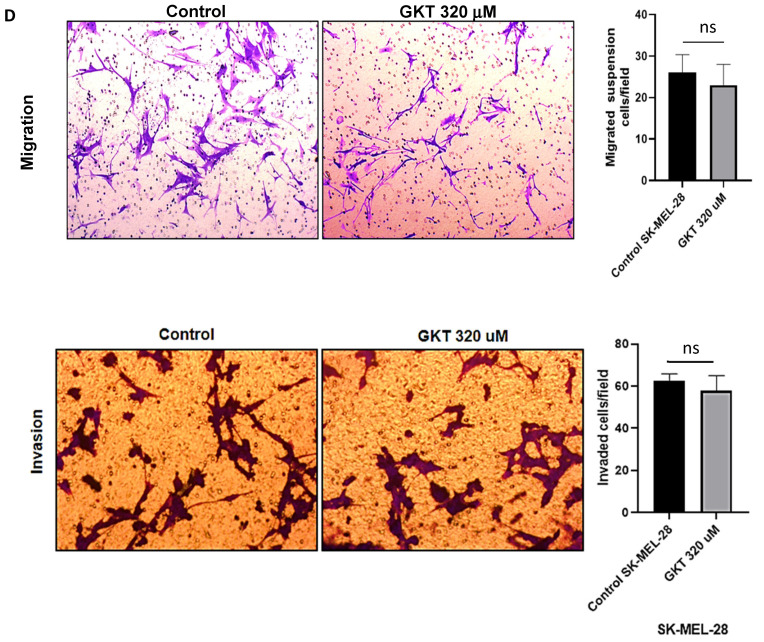
NOX4 and anoikis resistance in SK-MEL-28 cells. (**A**) NOX4 expression in SK-MEL-28 cells. Western blot analysis was conducted to assess NOX4 expression in SK-MEL-28 melanoma cells. The blot shown is representative of three independent experiments. (**B**) Cell viability assay for SK-MEL-28 cells maintained in suspension for 48 h and treated with increasing concentrations of GKT136901. The number of viable cells was determined by the Trypan Blue exclusion assay and expressed as mean ± SD in three independent experiments. Formation of multicellular aggregates of SK-MEL-28 cells kept in suspension and treated with GKT136901 (320 μM) for 48 h is shown (*n* = 3). (**C**) Expression levels of FAK, p-FAK Tyr397, ERK, p-ERK, and MMP-9 were determined by Western blot analysis. The blot images shown are representative of three independent experiments. When applicable, densitometric analysis was expressed as mean ± SD (*n* = 3). (**D**) Cell migration and invasion assays were performed using Transwell chambers with 8 μm pore-size membranes. After the experimental period, cells that migrated or invaded to the lower surface of the membrane were fixed, stained with crystal violet, and quantified. Representative images are shown, and results are expressed as mean ± SD from three independent experiments (*n* = 3), *** *p* < 0.05.

NOX4 was expressed in SK-MEL-28 cells (Figure 6A). The role of NOX4 in H_2_O_2_-mediated anoikis resistance of SK-MEL-28 cells was examined by exposing cells in suspension to increasing concentrations of GKT136901, an NOX4 inhibitor [34]. SK-MEL-28 cells in suspension treated with 320 µM, GKT136901, resulted in the inhibition of the expression of NOX4 (Figure 6A) with reduction on cell viability, and impairment on their ability to form multicellular aggregates (Figure 6B). GKT136901 decreased FAK expression, FAK-Tyr397 phosphorylation (Figure 6C), and ERK1/2 phosphorylation, resulting in the inhibition of ERK1/2 MAPK signaling, while MMP-9 expression slightly increased. Despite these molecular changes, no significant difference was observed in the cell migration or invasion assays, indicating that GKT136901 does not substantially affect the migratory or invasive capacities of SK-MEL-28 cells in suspension (Figure 6D).

### 3.4. Enrichment Pathway Analysis of Protein–Protein Interaction Networks

Given the importance of NOS3 and NOX4 in melanoma progression, we conducted an in silico analysis to investigate the interactions of NOS3 and NOX4 with other components of the signaling network related to anoikis resistance and characterized in the present study.

**Figure 7:**
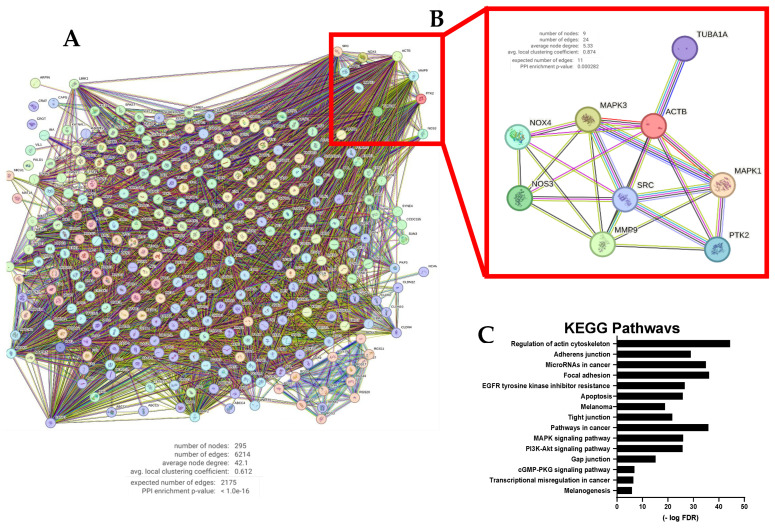
Analysis of the protein–protein interaction network and functional pathways associated with FAK-NOX4-NOS3-Src-β-actin-α-tubulin-ERK 1/2-MMP-9. (**A**) Complete interaction map with more than 300 proteins (PPI enrichment *p*-value < 1.0 × 10^−16^), including FAK-NOX4-NOS3-Src-β-actin-α-tubulin-ERK 1/2-MMP-9, generated using the String 10.5 program http://string-db.org (accessed on 27 April 2026) to mimic the interaction of this group under in vivo conditions. (**B**) Highlight of the FAK-NOX4-NOS3-Src-β-actin-α-tubulin-ERK 1/2-MMP-9 group within the main protein network. The network nodes represent the proteins: FAK-NOX4-NOS3-Src (central hub of the interaction network)—β-actin (central hub of the interaction network)—α-tubulin—ERK 1/2—MMP-9 (central hub of the interaction network). Different colored lines display the predicted functional links: experimentally determined interactions are shown in pink; interactions obtained from databases are in blue; co-expression is represented in black; and text mining is in green. The analysis showed: nine nodes with an average degree of 5.33; number of edges: 24; expected number of edges: 11; average local clustering coefficient: 0.874; PPI enrichment *p*-value: 0.000282. (**C**) Main anoikis-related processes in tumor cells ranked by enriched proteins in KEGG pathway analysis. The bar graphs show the –log of the false discovery rate (FDR).

The interactions between NOS3, NOX4, FAK (PTK2), Src, α-tubulin (TUBA1A), β-actin (ACTB), ERK 1/2 (MAPK3 and MAPK1 respectively), and MMP-9 were incorporated into a broad scope of 303 proteins (PPI enrichment *p*-value < 1.0 × 10^−16^) (Figure 7A) in the database http://string-db.org (accessed on 27 April 2026) to investigate the interaction of this group in a situation mimicking the in vivo condition. In this context, β-actin, MMP-9, and Src were determined as potential interaction centers with the remaining six proteins.

The network connecting these nine proteins of interest presented 24 edges, a number higher than the 11 edges predicted by the database. This suggests that the nine proteins interact with each other to a greater extent than expected for a random group of proteins of the same size and degree of distribution extracted from the genome. The *p*-value calculated for PPI enrichment was 0.000282, which is statistically significant (*p* < 0.001). This indicates that the nine proteins exhibit a high degree of biological connectivity as a group, even when in the presence of several other proteins (Figure 7B).

The KEGG analysis showed more than 100 pathways, among which 15 were selected based on false discovery rate (FDR) and Strength (S) values (Figure 7C). These analyses showed enrichment especially for genes of the adherent junction pathway (FDR = 1.09 × 10^−29^; S = 1.46), melanoma (FDR = 1.56 × 10^−19^; S = 1.31), regulation of actin cytoskeleton (FDR = 3.82 × 10^−45^; S = 1.24), apoptosis (FDR = 1.39 × 10^−26^; S = 1.23) and focal adhesion (FDR = 6.87 × 10^−37^; S = 1.21).

**Figure 8:**
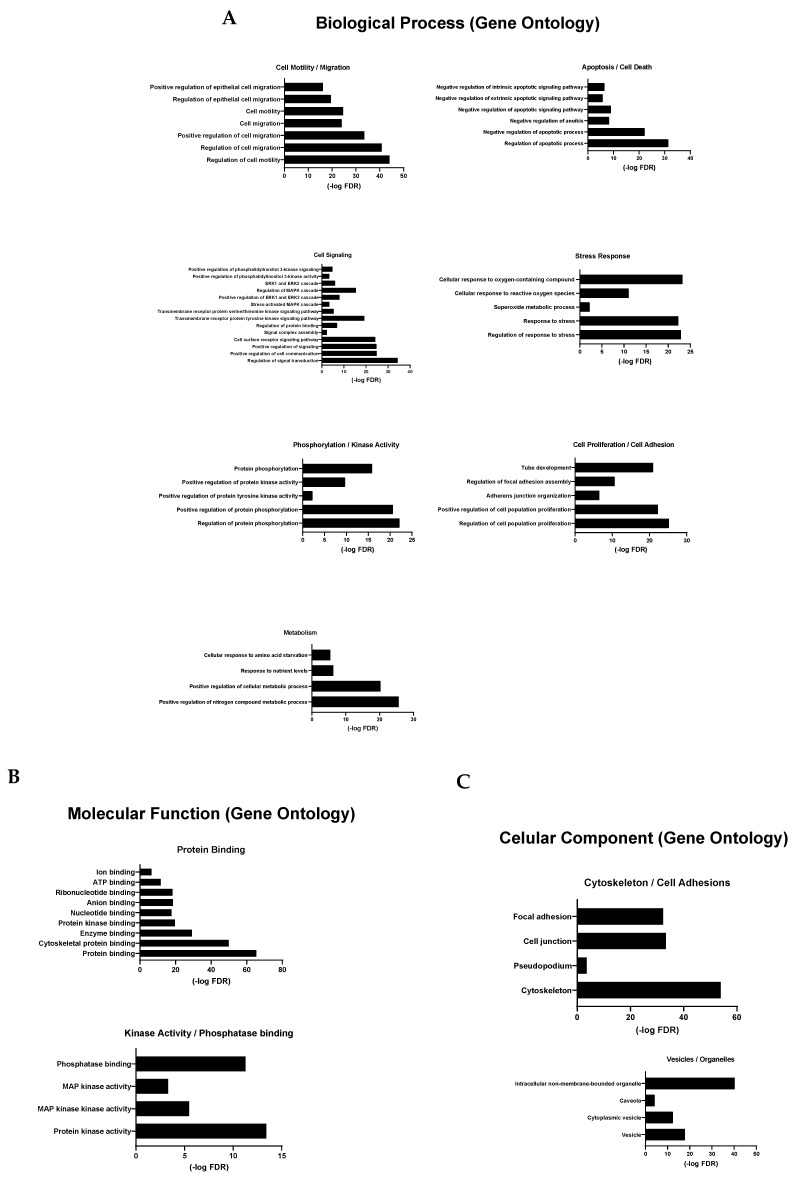
Analysis of enriched pathways in Gene Ontology (GO) associated with FAK-NOX4-NOS3-Src-β-actin-α-tubulin-ERK 1/2-MMP-9. (**A**) Biological processes. (**B**) Molecular functions. (**C**) Cellular components. Bar graphs show the −log of the false discovery rate (FDR).

The Gene Ontology (GO) assessment revealed over 100 biological processes (Supplementary Material S1—Enrichment Process), of which 46 were selected based on higher false discovery rate (FDR) and Strength (S) values and divided into categories of Cell Motility/Migration, Apoptosis/Cell Death, Cell Signaling, Stress Response, Phosphorylation/Kinase Activity, Cell Proliferation/Cell Adhesion, and Metabolism (Figure 8A). Among these, the processes that stood out the most were negative regulation of anoikis (FDR = 6.09 × 10^−9^; S = 1.5), regulation of focal adhesion assembly (FDR = 2.32 × 10^−11^; S = 1.2), adherens junction organization (FDR = 3.02 × 10^−7^; S = 1.15), cellular response to amino acid starvation (FDR = 4.03 × 10^−6^; S = 1.09), ERK1 and ERK2 cascade (FDR = 1.01 × 10^−6^; S = 1.08), positive regulation of epithelial cell migration (FDR = 8.19 × 10^−17^; S = 1.06), and cellular response to reactive oxygen species (FDR = 9.13 × 10^−12^; S = 1.03) and regulation of epithelial cell migration (FDR = 3.01 × 10^−20^; S = 1.0).

GO analysis showed more than 50 molecular functions (Supplementary Material S2—Enrichment Function), and 12 were selected based on the criteria described above (Figure 8B). Among these, the functions that stood out the most were MAP kinase activity (FDR = 0.00050; S = 1.35), phosphatase binding (FDR = 5.28 × 10^−12^; S = 0.91), cytoskeletal protein binding (FDR = 1.24 × 10^−50^; S = 0.83), and protein kinase binding (FDR = 1.95 × 10^−20^; S = 0.72).

Following the analysis of molecular functions, the GO analysis also identified more than 100 cellular components (Supplementary Material S3—Enrichment Components), and 10 were selected using the same criteria mentioned above (Figure 8C). Of these, the components that stood out the most were pseudopodium (FDR = 0.00026; S = 1.27), focal adhesion (FDR = 5.43 × 10^−33^; S = 0.96), caveolae (FDR = 8.65 × 10^−5^; S = 0.96), cytoskeleton (FDR = 1.30 × 10^−54^; S = 0.63), and cell junction (FDR = 5.20 × 10^−34^; S = 0.56).

The data derived from bioinformatics simulations of protein interaction networks presented here strongly suggest that all proteins characterized in this study actively participate in the signaling network related to anoikis resistance in melanomas.

## 4. Discussion

Resistance to anoikis is a critical determinant of tumor progression and metastatic colonization [4,5,7]. In this context, metastatic SK-MEL-28 cells exhibited greater resistance to anoikis compared to primary A375 cells. This phenotype has been consistently associated with activation of pro-survival signaling pathways, including those mediated by FAK/Src and PI3K/AKT, which promote cytoskeletal organization, integrin signaling, and suppression of apoptosis. Other tumor types, including prostate and triple-negative breast cancer, where metastatic cells display enhanced anoikis resistance often associated with the expression of proteins such as Talin1, which regulates focal adhesion dynamics [35,36,37].

Exposure of A431 human squamous carcinoma cells to high concentrations of NO donors induces detachment and apoptosis [38], whereas in HeLa human cervical cancer cells and murine melanoma cells, NO promotes resistance to anoikis [21], highlighting its dual role. This behavior is largely dependent on NO concentration and the cellular redox environment. At lower concentrations, NO promotes pro-survival signaling through redox-dependent mechanisms, whereas at higher concentrations, it induces nitrosative stress and apoptosis [18,39].

Human breast carcinoma MDA-MB-231 cells in suspension, subjected to low-intensity flow shear stress, become anoikis-resistant upon enhanced intracellular production of NO and ROS [40].

A375 cells generated higher intracellular NO levels compared to SK-MEL-28 cells, suggesting a differential redox regulation between primary and metastatic cells. While NO can contribute to cytoprotection by modulating mitochondrial function and inhibiting caspase activation through S-nitrosylation, excessive NO production may lead to mitochondrial dysfunction and enhanced susceptibility to apoptosis [39]. This is consistent with the increased sensitivity of A375 cells to prolonged exposure to the NO donor Detano (Figure 2C). In contrast, lower intracellular NO production by SK-MEL-28 cells in suspension results in greater resistance to increasing NO concentration (Figure 2C).

Endogenous NO production in melanoma cells is associated with the expression and activity of the NOS [41,42]. The expression levels of NOS2 and NOS3 are higher in A375 cells than in SK-MEL-28 cells in suspension. This difference may explain the elevated intracellular NO levels observed in A375 cells compared to those in SK-MEL-28 cells.

Knockdown of either isoform in A375 cells kept in suspension did not affect their ability to resist anoikis, suggesting that partial elimination of one isoform is compensated by the expression of the other isoform. The lack of a significant phenotype in NOS2-knockdown A375 cells (Figure 3A) and in NOS3-knockdown A375 cells (Figure 3B) highlights the importance of NO-mediated survival signaling in these cells. The presence of multiple NOS isoforms likely provides a compensatory reservoir of NO, ensuring survival during cell detachment. This redundancy is only overcome when all isoforms are simultaneously inhibited by L-NAME, as evidenced by the significant induction of anoikis (Figure 4).

Since L-NAME preferentially inhibits constitutive NOS isoforms [43], particularly NOS3, these findings suggest that NOS3-derived NO plays a key role in maintaining survival under anchorage-independent conditions. In addition, inhibition of NO production has been associated with inhibition of pro-survival pathways and increased apoptotic susceptibility, emphasizing its role in tumor cell survival [44,45,46,47].

The formation of multicellular aggregates, a key adaptive response to prevent anoikis, depends on cytoskeletal integrity [48]. The formation of the aggregates is dependent on β actin expression levels [49]. In addition, anoikis resistance is directly linked to the ability of cells to attach to a growth-permissive surface after being in suspension for extended periods. In breast cancer cells, attachment is aided by tubulin-based plasma membrane extensions [50]. Adequate expression levels of β-actin and α-tubulin are necessary for conferring resistance to anoikis in human prostate cancer cells [51].

NO promotes anoikis resistance through regulation of cytoskeletal organization and survival signaling [52]. In A375 cells, NO-dependent regulation of β-actin and α-tubulin most likely contributed to the maintenance of these structures, facilitating cell–cell interactions and survival in suspension.

FAK phosphorylation at Tyr397, a critical event for its activation [53], was reduced following L-NAME treatment, directly linking NO availability to FAK signaling [20,22]. Since FAK is a central mediator of integrin-dependent survival pathways, its inhibition provides a mechanistic explanation for the reduced anoikis resistance observed upon NOS inhibition.

Downstream of FAK, ERK signaling was strongly dependent on both adhesion and NO availability. ERK1/2 phosphorylation was elevated in adherent cells and significantly reduced in suspended cells, reflecting a disruption of integrin-mediated signaling [54]. This correlated with increased apoptotic markers, confirming the role of ERK1/2 in suppressing anoikis. L-NAME treatment further decreased ERK1/2 phosphorylation in suspended cells, demonstrating that endogenous NO sustains ERK1/2 activation under anchorage-independent conditions.

Decreased ERK1/2 phosphorylation following NOS inhibition emphasizes the role of NO in sustaining pro-survival pathways, including the FAK/Src and the MAPK/ERK1/2 signaling pathways [55,56]. In addition, ERK1/2 signaling regulates the expression of the matrix metalloprotease MMP-9, which is critical for tumor invasion and metastasis [57]. Therefore, modulation of ERK1/2 activity by NO directly impacts the invasive phenotype of melanoma cells. Functionally, these molecular alterations were accompanied by a significant reduction in the migratory and invasive capacities of A375 cells, following L-NAME treatment (Figure 4D), indicating that NO-dependent signaling is required for efficient cell motility and invasion. This is consistent with the role of FAK/ERK1/2 signaling in regulating focal adhesion turnover and actin cytoskeleton remodeling, processes essential for migration [53,58].

While NO plays a central role in *anoikis* resistance in A375 cells, metastatic SK-MEL-28 cells rely on alternative redox-based mechanisms. Increased production of H_2_O_2_ occurred in SK-MEL-28 cells suspension (Figure 5B). Hydrogen peroxide and other ROS activate signaling pathways, including MAPK/ERK and FAK/Src, which promote survival and tumor progression [59].

In contrast to A375 cells, exposure to L-NAME at the concentrations used in this study did not affect the viability of suspended SK-MEL-28 cells. This suggests that, although these cells express the three NOS isoforms, NO is not essential for their resistance to anoikis. In B16F10/Nex10C (from the primary site) and Nex8H (from the metastatic site) murine melanoma cells, bradykinin stimulation operates under spatial constraints and temporal regulation of signaling mediated by both NO and ROS [29]. In this study anoikis resistance requires NO in A375 cells and H_2_O_2_ in SK-MEL-28 cells.

In H460 lung cancer cells, treatment with low concentrations of H_2_O_2_ (25 μM) promoted resistance to anoikis [60]. SK-MEL-28 cells showed a high tolerance to exogenous H_2_O_2_, maintaining viability even at elevated concentrations of the oxidant, although their ability to reattach was impaired (Figure 5C,D). This suggests that endogenous ROS production, rather than exogenous exposure, is crucial for anoikis resistance.

NOX4, a major source of H_2_O_2_ in melanoma, is constitutively active and regulated by its expression levels [26,61]. The enzyme primarily produces H_2_O_2_ with a significantly lower production of O_2_^−^ [61]. NOX4 has its expression elevated in metastatic melanoma, protecting the highly metastatic MV3 human melanoma cell line against apoptosis by activating the signaling axis FAK-Src kinase [27].

Adhesion to a surface permissive to growth was impaired in SK-MEL-28 cells maintained in suspension in serum-free medium and treated with the NOX4 inhibitor GKT136901 [34]. However, migration and invasion capacities of these cells were not affected by the treatment with GKT136901, suggesting that ROS regulate different signaling pathways associated with adhesion, migration, and invasion.

NOX4-derived H_2_O_2_ contributed to anoikis resistance in SK-MEL-28 cells in suspension. Inhibition of NOX enzymes using GKT136901 reduced cell viability, supporting the role of ROS in maintaining survival.

## 5. Conclusions

Early observations account for H_2_O_2_ stimulation of phosphorylation of FAK at Tyr397, which results in enhanced cell migration of endothelial and intestinal epithelial cells [62,63]. Suspended SK-MEL-28 cells treated with GKT136901 inhibited the formation of multicellular aggregates and phosphorylation of FAK at Tyr397. These findings suggest that, like NO in suspended A375 cells, H_2_O_2_ can modulate FAK phosphorylation at Tyr397 and assemble a signaling complex associated with resistance to anoikis.

**Figure 9:**
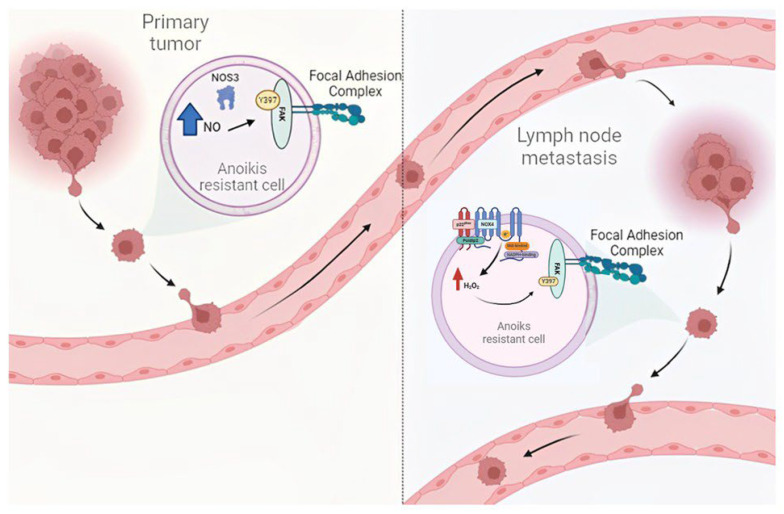
Redox-regulated activation of focal adhesion kinase (FAK) is associated with anoikis resistance in human melanoma. Phosphorylation on Tyr397 is associated with FAK activation. Activation is mediated by NO in melanomas at the primary site, represented in this study by A375 cells, and by H_2_O_2_ in metastatic melanomas, represented here by SK-MEL-28 cells.

According to the PPI model (Figure 7 and Figure 8), which was constructed using data from this and previous studies [21,22], it is proposed that this signaling complex consists of the oxidant-generating enzymes NOS3 and NOX4, the non-receptor tyrosine kinases FAK and Src, the ERK1/2 MAP kinases, the metalloprotease MMP-9, and the structural proteins α-tubulin and β-actin. While β-actin is the hub of the signaling complex, FAK may act as a redox switch in the complex, activated by NO/NOS3 or H_2_O_2_/NOX4 depending on the stage of development of the melanoma (Figure 9). These results highlight a shift in redox dependency of anoikis resistance in melanoma cells, where cells at the primary site rely more on NO-mediated signaling, while metastatic cells depend on ROS-mediated signaling events.

Given the importance of resistance to anoikis in the metastatic process, understanding the mechanisms underlying the redox regulation of anoikis resistance is pivotal to establish new and efficient therapeutic strategies to prevent melanoma metastasis.

## Figures and Tables

**Table 1:** **sgRNAs designed for CRISPR-mediated repression of NOS3**.

sgRNA NOS3 I-BOTTOM.	5′-AAACGTAGTTTCCGTGGAAATACC-3′
sgRNA NOS3 II-TOP	5′-CACCGCCTCCCAGTTCTTCACACGA-3′
sgRNA NOS3 II-BOTTOM	5′-AAACTCGTGTGAAGAACTGGGAGGC-3′

## Data Availability

The data sets used and/or analyzed during the current study are available from the corresponding author.

## Supplementary Materials

The following supporting information can be downloaded at: https://www.mdpi.com/article/10.3390/antiox15060740/s1, Figure S1: A375 and SK-MEL-28 adhered, Supplementary Material S1—Enrichment Process, Supplementary Material S2—Enrichment Function, and Supplementary Material S3—Enrichment Components).
