# Supplementary material for: Nitric Oxide, Reactive Oxygen Species, and Focal Adhesion Kinase Mediate Anoikis Resistance in A375 and SK-MEL-28 Human Melanoma Cells
Source: Antioxidants (Basel). 2026 Jun 10;15(6):740. doi: 10.3390/antiox15060740 (PMC13295398; doi:10.3390/antiox15060740)
Supplement: Supplementary file 1 [file antioxidants-15-00740-s001.zip › Figure S1.pptx]

## Slide 1
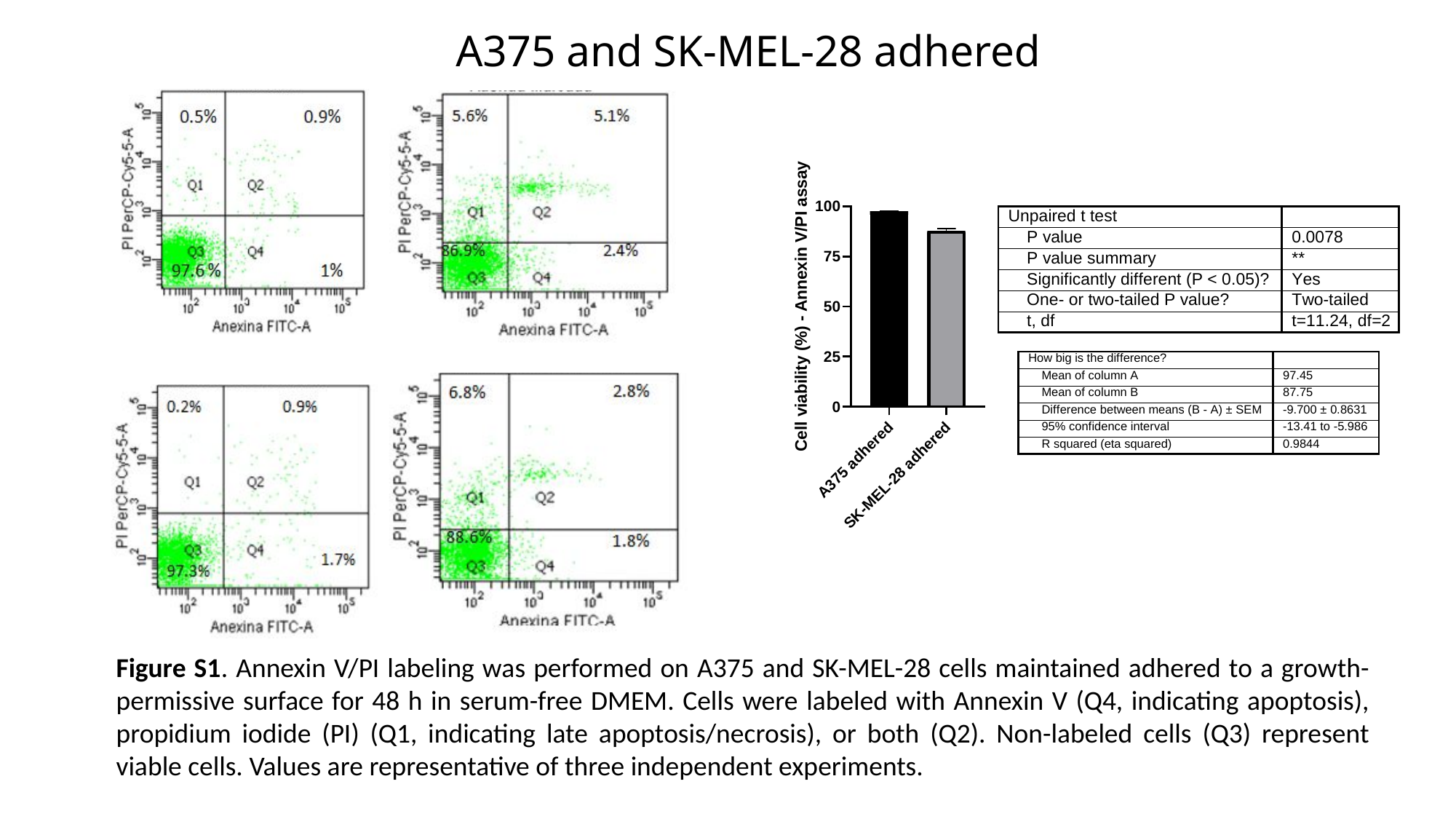

# A375 and SK-MEL-28 adhered
Figure S1. Annexin V/PI labeling was performed on A375 and SK-MEL-28 cells maintained adhered to a growth-permissive surface for 48 h in serum-free DMEM. Cells were labeled with Annexin V (Q4, indicating apoptosis), propidium iodide (PI) (Q1, indicating late apoptosis/necrosis), or both (Q2). Non-labeled cells (Q3) represent viable cells. Values are representative of three independent experiments.
